# Successful rechallenge with ceritinib after leukocytoclastic vasculitis during ceritinib treatment for non-small cell lung cancer harboring the EML4-ALK fusion protein

**DOI:** 10.18632/oncotarget.24765

**Published:** 2018-04-13

**Authors:** Tamio Okimoto, Yukari Tsubata, Takamasa Hotta, Megumi Hamaguchi, Takae Okuno, Yohei Shiratsuki, Akari Kodama, Mika Nakao, Yoshihiro Amano, Shunichi Hamaguchi, Noriaki Kurimoto, Reiko Tobita, Takeshi Isobe

**Affiliations:** ^1^ Department of Internal Medicine, Division of Medical Oncology and Respiratory Medicine, Shimane University Faculty of Medicine, Shimane, Japan; ^2^ Department of Dermatology, Shimane University Faculty of Medicine, Shimane, Japan

**Keywords:** alectinib, anaplastic lymphoma kinase, ceritinib, leukocytoclastic vasculitis, non-small cell lung cancer

## Abstract

Anaplastic lymphoma kinase (ALK)-tyrosine kinase inhibitors (TKIs) dramatically improve progression-free survival compared to cytotoxic agents. It is therefore important to manage patients with ALK-TKIs until drug resistance occurs. Leukocytoclastic vasculitis (LCV) is a rare complication during cancer treatment and is associated with a variety of factors. Currently, it is unclear whether we should withdraw a treatment when drug-induced LCV develops.

We report a 40-year-old man with advanced pulmonary adenocarcinoma harboring the EML4-ALK fusion protein who developed LCV during ceritinib treatment. Four weeks after withdrawing ceritinib, we could successfully perform rechallenge with ceritinib at the normal dose. Rapid and massive tumor apoptosis due to ceritinib treatment may lead to neoantigen release and immune complexes deposition.

To the best of our knowledge, we report the first case of LCV in a patient during ALK-TKI treatment. Following this occurrence, we were able to successfully perform rechallenge with ceritinib. Therefore, key drugs used in a patient's treatment regimen should not be discontinued without careful evaluation, and we should also consider the possibility of rechallenge.

## INTRODUCTION

Lung cancer is the leading cause of death due to cancer. Molecular testing revealed the EML4-ALK fusion protein in approximately 5% of patients with non-small cell lung cancer [1, 2]. As anaplastic lymphoma kinase (ALK)-tyrosine kinase inhibitors (TKIs) dramatically improve progression-free survival compared to cytotoxic agents [3–6], ALK-TKIs are commonly used for ALK fusion protein-positive non-small cell lung cancer unless the tumor becomes resistant to the drug.

Leukocytoclastic vasculitis (LCV) is a rare complication during cancer treatment. It is associated with chronic infection, drugs and para-neoplastic syndrome. When diagnosing LCV during cancer treatment, it is very important to determine whether the vasculitis is associated with cancer drugs, especially driver mutation targeted drugs, which are the most important drugs for the patients, and whether rechallenge with the drug can be done safely. Some reports have shown safe rechallenge with epidermal growth factor receptor (EGFR)-TKIs after LCV that occurred during EGFR-TKI therapy. However, there are no previous reports of LCV associated with ALK-TKIs; this is the first report of safe rechallenge with ceritinib after LCV.

## CASE PRESENTATION

**Figure 1:**
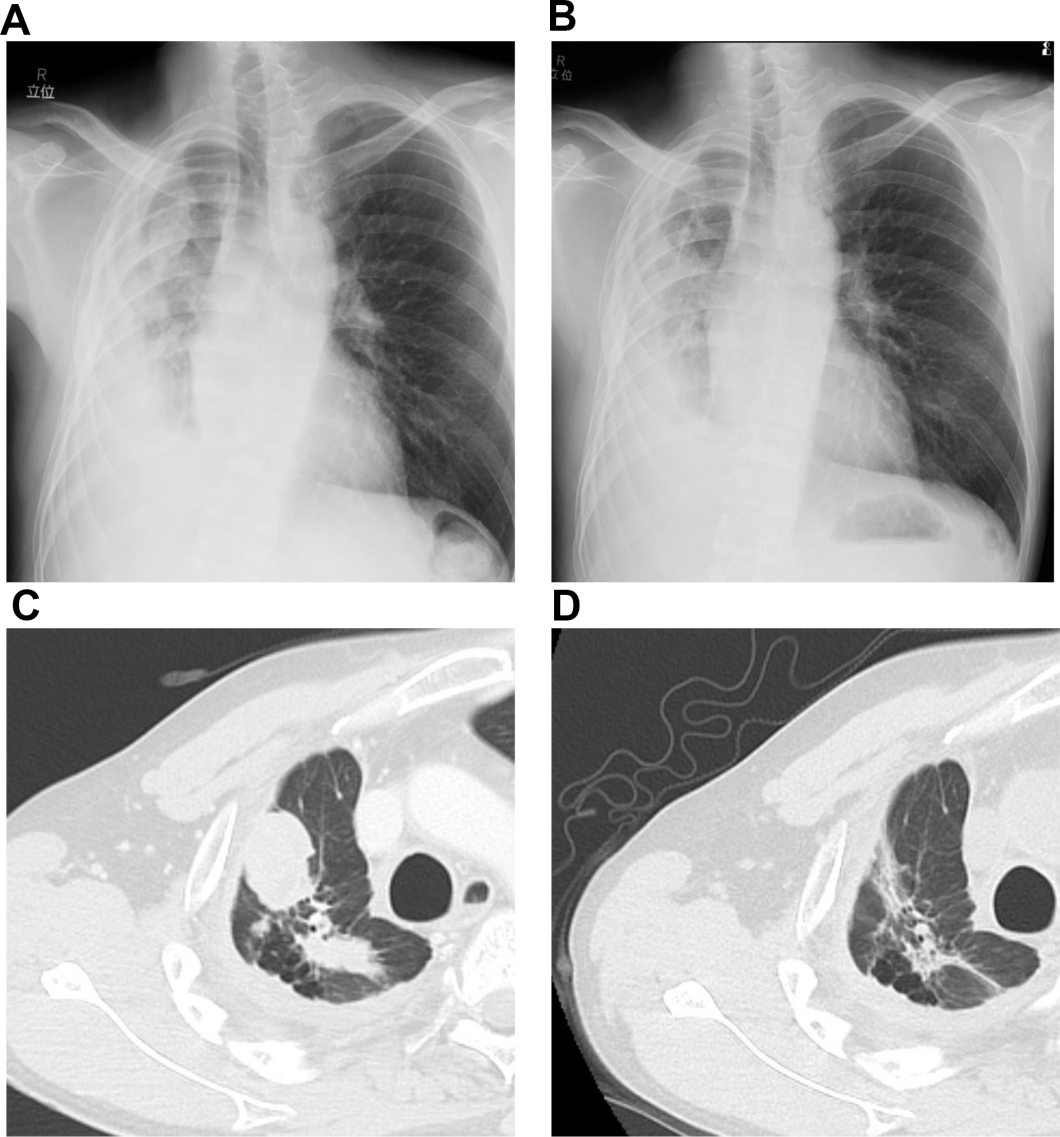
(**A**, **C**) Chest radiograph and computed tomography scan before starting ceritinib. (**B**, **D**) Chest radiograph and computed tomography scan after ceritinib treatment.

**Figure 2:**
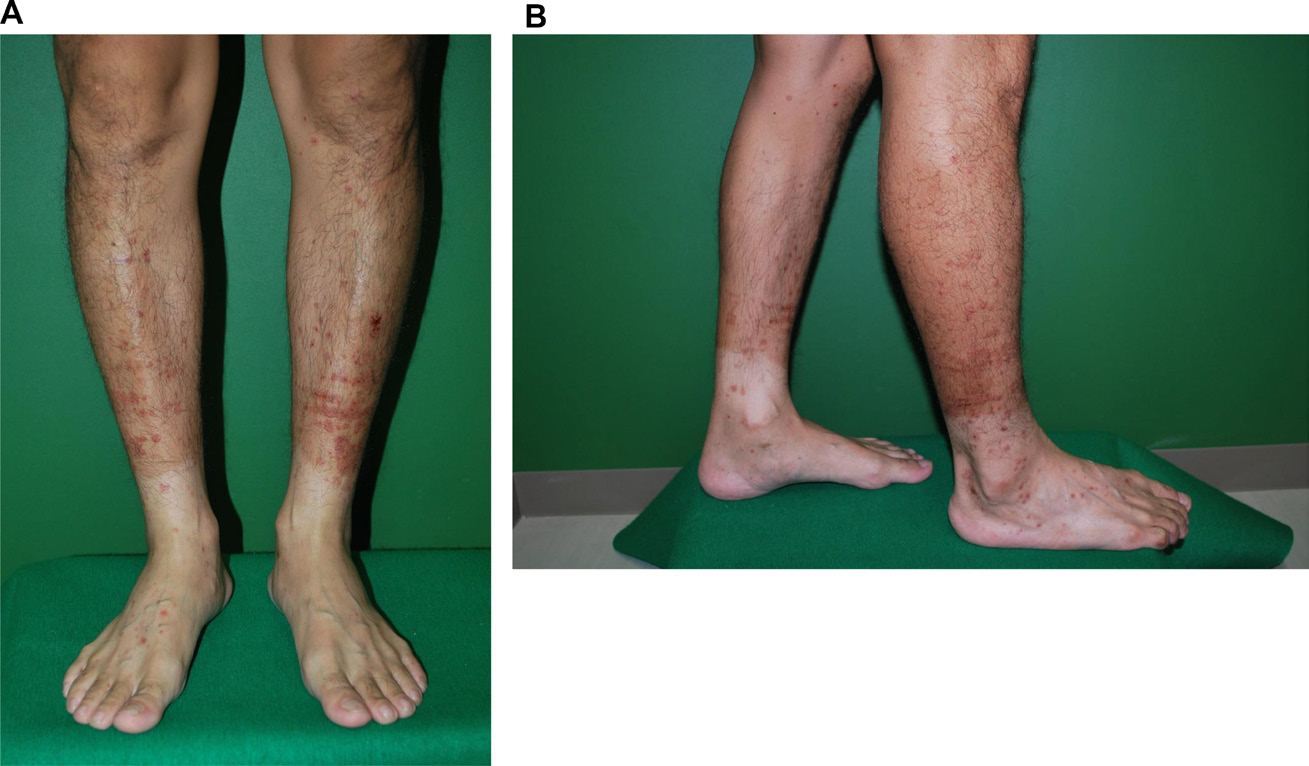
(**A, B**) Palpable purpura on the lower extremities.

**Figure 3:**
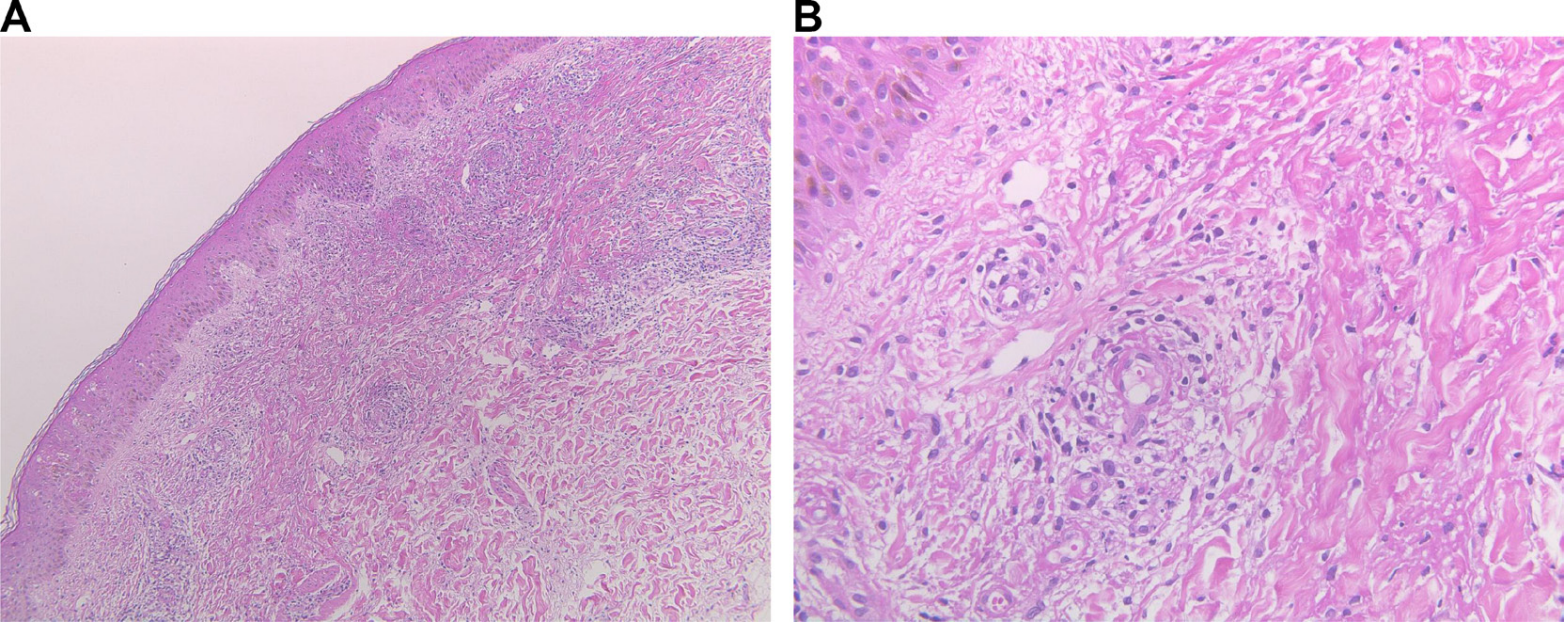
Pathological findings of the skin biopsy sample demonstrating leukocytoclastic vasculitis (**A**) Hematoxylin and eosin staining (×40). (**B**) Hematoxylin and eosin staining (×400).

A 40-year-old Japanese man presented to our hospital with pulmonary adenocarcinoma. He had been diagnosed with adenocarcinoma 2 years ago, and RNA sequencing revealed the presence of the EML4-ALK fusion protein. When he was diagnosed, he rejected all our recommendations for chemotherapy including ALK-TKI and he stopped his annual visits to our clinic. Six months after the initial diagnosis, he presented to our hospital with dyspnea due to cardiac tamponade and pleural effusion. He received pericardiocentesis and thoracentesis, and he agreed to start crizotinib treatment (250 mg, twice daily). One month after the initiation of crizotinib, a computed tomography (CT) scan showed decreased pleural and pericardial effusion and shrinkage of the primary lesion. Ten months after the initiation of crizotinib, progressive disease (PD) was detected considering the enlargement of the primary lesion. Therefore, alectinib (300 mg, twice daily) was administered as second-line treatment, and a CT scan showed a partial response. Fourteen months after the initiation of alectinib, PD was detected again as an enlargement of the primary lesion. We continued alectinib until ceritinib was launched in Japan. One month after the PD detection, we switched to ceritinib (750 mg once daily) and the CT scan showed a partial response (Figure 1). Ten days after initiating ceritinib, palpable purpura was observed (Figure 2). The test results for myeloperoxidase-anti-neutrophil cytoplasmic antibody (MPO-ANCA) and proteinase 3 anti-neutrophil cytoplasmic antibody (PR3-ANCA) were negative. Skin biopsy showed LCV (Figure 3A–3B). IgA deposition was negative. Because ceritinib was the only drug we started one month before the onset of LCV, we suspected this was associated with ceritinib. We recommended withdrawal of ceritinib to the patient, however, he rejected it and therefore we continued ceritinib for another month. During this period, the purpura gradually improved, although, the patient caught a common cold and proteinuria was observed. We could not continue the drug as the patient was at risk for developing kidney vasculitis and he agreed. We therefore withdrew only ceritinib until the purpura improved. Four weeks after withdrawal, we confirmed the disappearance of the purpura and performed a rechallenge with ceritinib at the usual dose (750 mg once daily) without using any immunosuppressants. Although slight re-exacerbation of purpura was observed, it disappeared quickly, and the tumor response was maintained for 9 months after the rechallenge. Now he has been receiving carboplatin (area under the concentration-time curve 5), pemetrexed (500 mg/m^2^) and bevacizumab (15 mg/kg) therapy without relapse of LCV.

## DISCUSSION

This case had two important clinical characteristics. First, this is the first case of LCV during ALK-TKI treatment. Second, we safely performed rechallenge with ceritinib after LCV during ceritinib treatment.

LCV is vasculitis of the small vessels in which the inflammatory infiltrate is composed of neutrophils that release nuclear debris, i.e., leukocytoclasia. The development of LCV is associated with both immune complex deposition and hypersensitivity to a suspected drug. Examples of immune complex deposition related to disease include cryoglobulinemic vasculitis related to hepatitis C, systemic lupus erythematosus, IgA vasculitis, and paraneoplastic syndrome. Several potential reasons have been proposed for the mechanism of paraneoplastic vasculitis [7]. One hypothesis is that abnormal production of antibodies and tumor neoantigens lead to the formation of immune complexes that deposit within blood vessel walls. In this case, it is plausible that rapid and massive tumor apoptosis due to ceritinib treatment led to neoantigen release and immune complexes deposition. The purpura gradually improved after the onset of LCV despite continuation of ceritinib. It was possibly associated with immune complex clearance through blood vessels. In addition, a slight re-exacerbation and quick disappearance of purpura, which were observed after rechallenge of ceritinib. may have been associated with increased tumor burden due to withdrawal of ceritinib for 4 weeks and rapid and massive apoptosis due to ceritinib rechallenge. LCV may be a putative manifestation of drug efficacy. Brandi *et al.* also reported on this possibility [8].

As examples of hypersensitivity to a drug related to LCV, propylthiouracil, hydralazine, colony-stimulating factors, and allopurinol have been most often implicated as a causative drug for drug-induced LCV [9–12]. Although the mechanism of LCV development remains unclear, one hypothesis suggests that activated neutrophils in the presence of hydrogen peroxidase release MPO from their granules, chemically transform the drug to an immunogenic product for T cells, which in turn activate B cells to produce ANCA [13]. That is why multispecific ANCA is common in drug-induced LCV unlike idiopathic autoimmune vasculitis [11, 14]. In some cases, vasculitis occurred after drug dosage increases and after rechallenge with the suspected drug [12]. In this case, we safely performed rechallenge with ceritinib and ANCA was negative, which suggests that this is a case of LCV not associated with hypersensitivity to ceritinib but associated with neoantigen release and immune complexes deposition.

Some LCV cases during non-small cell lung cancer (NSCLC) treatment have been reported to date (Table 1) [15–26]. In most cases, LCV developed 1–2 months after the initiation of EGFR-TKI and skin purpura improved within a month after withdrawal EGFR-TKI, similar to our case. Regarding EGFR-TKI treatment, 1 LCV case during gefitinib treatment for adenoid cystic carcinoma of the maxilla [27] and 2 LCV cases during erlotinib treatment for hepatocellular carcinoma were reported [8, 28]. However, in most of the cases, the dose of the suspected drug, gefitinib or erlotinib, was reduced [15, 17, 19, 20] or the drug was discontinued [16, 21], and in only 2 cases, successful rechallenge at a normal dose was reported [18, 19]. Regarding the cytotoxic drugs, pemetrexed, gemcitabine, etoposide, and docetaxel were reported to be a causative drug for LCV. Although significantly more patients have received cytotoxic chemotherapy than EGFR-TKIs, more LCV cases have been reported to date with use of EGFR-TKIs. This suggests an association between rapid tumor apoptosis as well as the EGFR-TKI's target (EGFR) and the development of LCV. If the LCV truly developed with hypersensitivity to the causative drug, it is very difficult to avoid LCV relapse only by reducing the dose or by providing intermittent administration of the drug. Both seropositive LCV cases [16] and seronegative LCV cases [20] during EGFR-TKI treatment have been reported. LCV cases during EGFR-TKI treatment include both paraneoplastic vasculitis and hypersensitivity related vasculitis. Ota *et al.* reported a LCV case during NSCLC treatment, in which LCV developed as a paraneoplastic vasculitis along with disease progression [26].

**Table 1:** Published cases of leukocytoclastic vasculitis cases during non-small cell lung cancer treatment

Age, gender	Drug	Time to onset	Treatment	Prognosis	Duration of the symptom	Rechallenge, dose	Author
68, Female	erlotinib	10 weeks	dose reduction, topical steroid	cure	unknown	Yes, reduced dose	Yuba *et al.* [15]
69, Female	erlotinib	8 weeks	withdrawal topical steroid	cure	2 weeks	No	Takahashi *et al.* [16]
78, Female	erlotinib	80 days	withdrawal	cure	2 weeks	Yes, reduced dose	Sawada *et al.* [20]
50, Female	erlotinib + bevacizumab	6 weeks	withdrawal	cure	7 weeks	Yes, reduced dose	Su *et al.* [17]
52, Female	gefitinib	2 months	topical steroid	cure	unknown	Yes, normal dose	Nozato *et al.* [18]
74, Female	gefitinib	1 month	withdrawal	cure	2 weeks	Yes, intermittently	Uchimiya *et al.* [19]
76, Female	gefitinib	2 months	withdrawal	cure	17 days	Yes, normal dose	Uchimiya *et al.* [19]
76, Female	gefitinib	2.5 months	withdrawal systemic steroid	cure	2 weeks	No	Kurokawa *et al.* [21]
68, Male	pemetrexed	5 weeks	withdrawal systemic steroid	cure	3 days	unknown	Lopes *et al.* [22]
45, Male	gemcitabine	6 weeks	withdrawal systemic steroid colchicine	cure	10 days	No	Voorburg *et al.* [23]
79, Male	gemcitabine + carboplatin	8 days	withdrawal systemic steroid diphenhydramine	cure	15 days	No	Corella *et al.* [24]
61, Male	etoposide	10 days	withdrawal	cure	unknown	unknown	Turken *et al.* [25]
50, Male	docetaxel	after 12 cycles	withdrawal systemic steroid	cure	promptly resolved	No	Ota *et al.* [26]

In most cases, leukocytoclastic vasculitis developed 1–2 months after the initiation of causative drug and skin purpura improved within a month after withdrawal and/or systemic steroid.

Drug-induced LCV is sometimes life-threatening if the suspected drug is continued [12]. It is very difficult but important to distinguish drug-induced vasculitis from paraneoplastic vasculitis. The interval between the first exposure and appearance of symptoms has been reported to be extremely variable (hours to years) [12]. Serological surveys, especially for ANCA, may help to distinguish between the types of vasculitis [11]. If the result for ANCA is negative, a rechallenge with the suspected drug should be considered.

## CONCLUSIONS

To the best of our knowledge, we report the first case of LCV during ALK-TKI treatment. However, after this occurrence, we were able to safely perform rechallenge with ceritinib. From this case, we learned that key drugs should not be discontinued without careful consideration, and we should contemplate the possibility of rechallenge.

## Abbreviations

EML4: Echinoderm microtubule-associated protein-like 4
ALK: Anaplastic lymphoma kinase
TKI: tyrosine kinase inhibitor
LCV: leukocytoclastic vasculitis
EGFR: epidermal growth factor receptor
PD: progressive disease
CT: computed tomography
MPO-ANCA: myeloperoxidase-anti-neutrophil cytoplasmic antibody
PR3-ANCA: proteinase 3 anti-neutrophil cytoplasmic antibody
